# Predicting of Effective Dose as Biomarker for Cytotoxicity Using Partial Least Square-Fourier Transform Infrared Spectroscopy (PLS_FTIR)

**Published:** 2015

**Authors:** Rezvan Zendehdel, Soheila Khodakarim, Farshad H. Shirazi

**Affiliations:** a *Department of Occupational Hygiene, School of Public Health, Shahid Beheshti University of Medical Sciences, Tehran, Iran. *; b *Department of Epidemiology, School of Public Health, Shahid Beheshti University of Medical Sciences, Tehran, Iran. *; c *Pharmaceutical Sciences Research Center, Shahid Beheshti University of Medical Sciences, Tehran, Iran. *; d *Department of Toxicology and Pharmacology, School of Pharmacy, Shahid Beheshti University of Medical Sciences, Tehran, Iran. *

**Keywords:** Cisplatin, HepG2, Fourier transform infrared, Partial least square regression, Toxicity bioassay

## Abstract

Toxicity bioassays are important tools to determine biological effects of chemical agents on species. The questions remained on, what effects have been imposed on each of the different molecular site of cells by chemical exposure and how to find a pattern for chemical toxicity.

To address the questions, HepG2 cell lines were exposed to the different concentrations of cisplatin for 24 hours to result cell mortality in the range of one to one hundred percent. Fourier Transform Infrared spectroscopy (FTIR) has been used in this study to analyze the chemical alterations on HepG2 cell line by cisplatin. Partial least square regression (PLS) analysis was then applied to the FTIR spectrum results to search for a biomarker peak and present the desire cellular effects of cisplatin. The comparison of cellular FTIR spectra after exposure to different concentrations of cisplatin confirmed the binding of cisplatin to DNA through direct interaction of platinum to guanine and thymine bases of DNA. Biochemical Index Spectra (BIS) were defined based on the differences between of normal and cisplatin exposed cells. Information from the BIS was subjected to PLS analysis to trigger any particular relationship between the toxicity spectral response and cisplatin concentration. This approach was capable of predicting the concentration of cisplatin for any particular effects observed in the cellular FTIR spectrum (R^2^ = 0.968 ± 0.037).

Our work supports the promises that, FTIR can demonstrate the trace of toxicity before the cells dies. Finally, PLS of FTIR data directly predicts the effective concentration of chemicals in particular cellular components.

## Introduction

Toxicity bioassays are important tools to develop the biological effect of chemical agents on cells. Most of the cytotoxicity tests were based on the ability of cells to continue the biological activity during the test and measures the criteria of cell growing or cell death. In general, these tests could neither qualify toxicity effect in different molecules of cells (1) nor predict dose quantity in the pattern of cell toxicity. 

Infrared spectroscopy, have been used to investigate biochemical composition of cells in early nineties (2). Later, FTIR research was used in a large number of different studies including the early diagnosis of malignant tissues (3, 4), asses of plants organs (5) and estimating stress in microorganisms (6). These literatures have presented the potential application of FTIR technique for the detection of cellular stress or alterations with a good sensitivity. Several cluster analysis such as principal component analysis (PCA), artificial neural network (ANN) and genetic algorithms (GA) were used to analyze the FTIR spectra (-). Some authors challenged with machine learning and boosting support vector regression analysis to quantify the FTIR spectral data(10, 11). In some cases, the variation of the ratios between the areas of the peaks have accurate perturbation in spectral data (12).

Cisplatin is a cytotoxic agent that is used for treatment of many cancer types including testicular, ovarian, cervical, head and neck, non-small cell lung and lymphoma (13). There are many theories to explain platinum cytotoxicity, including interstrand and intrastrand cross-links with nitrogenous bases of deoxyribonucleic acids (-). These interactions causes error in DNA synthesis and alterations in cellular transcription (18, 19). Interaction of cisplatin with proteins and lipids was also believed to be responsible for the cytotoxicity of this cancer chemotherapy agent (20, 21). The effect of cisplatin on different molecular sites of cells provide a chance to apply a model to be useful for the understanding and even prediction of its cellular toxicity bioassay.

In the present study, cisplatin interaction with human hepatocarcinoma HepG2 cell line was investigated using FTIR-based assay. Different cisplatin concentrations were analyzed to study the binding properties of cisplatin to cells. This effort demonstrate biochemical change index which can be used for platinum cytotoxicity activity. We proposed a PLS method on FTIR spectrum data to predict effective concentration pattern of cisplatin toxicity in HepG2 cell line.

## Experimental

*Cell line*

Human hepatocarcinomacell line (HepG2) was obtained from Pasture Institute National Cell Bank of Iran (Tehran, Iran). Cell line was grown in RPMI-1640 medium supplemented with 10% heat inactivated fetal bovine serum, antibiotics: penicillin, streptomycin (all chemicals from Sigma). Cells were maintained at 37 °C in humidified atmosphere containing 5% CO_2_. 

*Experimental strategy*

Cells were trypsinized from the original flask and seeded in 75 cm^2^ flasks with fresh medium to reach the logarithmic phase of growth curve. After that cells were washed twice with saline (0.9% NaCl), suspended and centrifuged at 1000 rpm for 10 min, then resuspended in saline to obtain a concentration of 10^7^cells/mL. Part of these cells were used for FTIR experiments using nine concentrations as is shown in Table 1 and about 0.1 mL for the clonogenic assay. Total of attached and detached cells in each flasks were collected after 24 hours exposure for FTIR spectroscopy. All experiments were repeated seven times for each concentration and all of these resulted data were used in mathematical calculations.

**Table 1:** Clonogenic assay based mortality induced by cisplatin at different concentrations.

	**Concentration(µg/mL)**	**Cisplatin mortality**
1	0	0%
2	0.125	24%
3	0.25	40%
4	0.5	67%
5	1	88%
6	1.5	92%
7	2	95%
8	3	98%
9	4	100%

*Clonogenic assay*

One hundred cells from the cell suspension was seeded in each well of six wells cell culture plates with 2 mL of fresh medium. Cells in each well were exposed to one of different concentrations of cisplatin as is listed in Table 1 for 24 hours. Media was changed with fresh media and incubated for 10 days. After that the wells were stained with crystal violet and colonies containing more than 50 cells were counted in each well. Survival curve was resulted from the percentage of colonies in each well exposed to cisplatin compare to the control wells. 

*Cell preparation for spectroscopy*

The following procedure was similarly applied for all cell line flasks. 10 μL of each cell suspension was placed on a zinc selenide sample carrier which was dehydrated in a vacuum cabin (0.8 bar) for approximately 4 min. These plates were then used for FTIR spectroscopy (22).

*FTIR spectroscopy*

For FTIR studies, thin dried films of cell suspensions were used on the Zinc selenide window by using a WQF-510 (Rayleigh Optics, China) spectrometer, equipped with a KBr beam splitter and a DLaTGS (deuterated Lantanide triglycine sulphate) detector. In each spectrum, 100 scans were collected at a resolution of 4 cm^-1 ^for every wave number between 400 and 4000 cm^-1^. Each single spectrum was baseline corrected and then normalized in order to have the range spanning from 0 to 1*.*

*Data analysis*

*Data set*

A total of 56 FTIR spectrums (7 spectra for each concentration as is listed in Table 1) have been taken in the range of 1000-3000 cm^-1^ and results were arranged in a data set for different analysis as are listed below.

*Partial least square regression*

Partial least square regression (PLS) is a common prediction model in the field of chemo metrics method. PLS is a multiple linear regression model that is related to other methods including principal components regression (23). Where Y is an output object with m variables, and X is the input matrix with p predictor variables. Partial Least Squares regression is based on the simultaneous decomposition of X and Y into latent variables (T) and associated loading vectors (Q). Regression is performed on these components, thus Y = TQ + E, where Q is a matrix of regression coefficients (loadings) for T (24). Here, X and Y are the independent variables, respectively. In this work, Y includes concentration of cisplatin, while the X block consists of y axis of FTIR data for Biochemical Index spectrum.

## Results and Discussion

**Figure 1:**
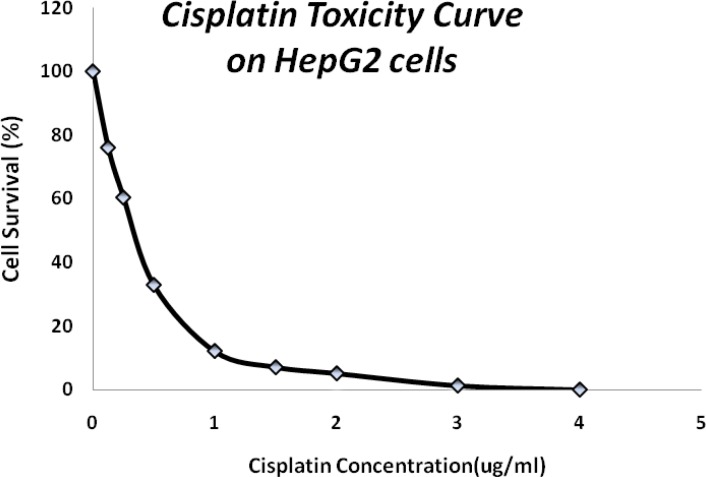
Cisplatin mortality in HepG2 cells following a 24 hours exposure time.

Clonogenic assay showed a lethal concentration fifty of 0.3 µg/mL and a sharp mortality effect of HepG2 cells up to 1.5 µg/mL of cisplatin exposure (Figure 1) which caused 90% cell death. 

*FTIR spectroscopy*

**Figure 2:**
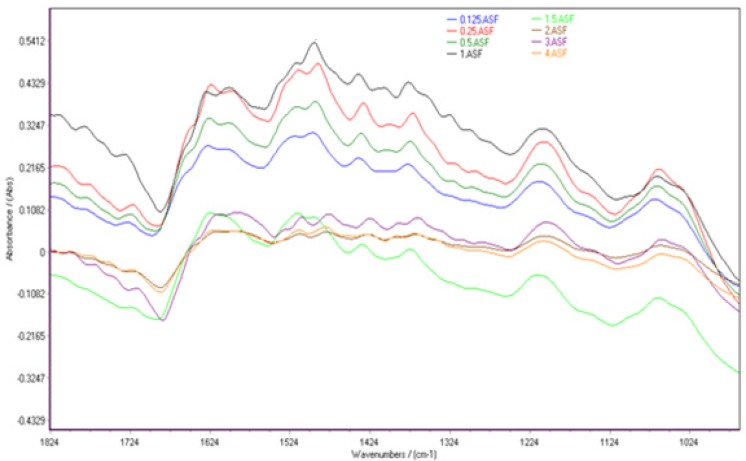
Spectral features of HepG2 cells after 24 hours exposure to the different concentrations of cisplatin in the FTIR spectral region of 1800-900 cm^-1^.

Spectral features of HepG2 cells in the range of 1800-900 cm^-1^ for the different concentrations of cisplatin are shown in Figure 2. The normalized FTIR spectra in this region showed alterations in different spectral areas. Comparison between spectra showed at least two areas of variation:

- Ring vibrations of nitrogenous bases (C=O, C=N stretching), PO_2_ stretching vibrations (symmetric and asymmetric) and deoxy-ribose stretching of DNA are appeared in the spectral region 1800 –700 cm^-1 ^(25). The vibrational bands of DNA at 1720, 1665, 1613 and 1499 cm^-1^ are assigned to guanine (G), thymine (T), adenine (A) and cytosine(C) nitrogenous bases, respectively (26). Cellular FTIR patterns are changed above 1.5 µg/mL as follows; guanine band at 1725cm^-1 ^shift to 1713 cm^-1^ and thymine band at 1665 cm^-1 ^shift toward a lower wave number at 1657 cm^-1^. These shifts can be related to platinum binding to N_7_ of Guanine and O_2_ of Thymine in DNA bases (14). Bands at 1228 and 1087 cm^-1^ demonstrate phosphate asymmetric and symmetric vibrations, respectively (27) (27). No major shift was observed for phosphate vibrations.
- Βeta-sheet structure spectra of proteins at 1639 (28) shifts to 1624 cm^-1^ up to 2 µg/mL. The observed spectral changes can be attributed to a coordination of the Pt cation and C–N group of polypeptide (29, 30).

**Figure 3:**
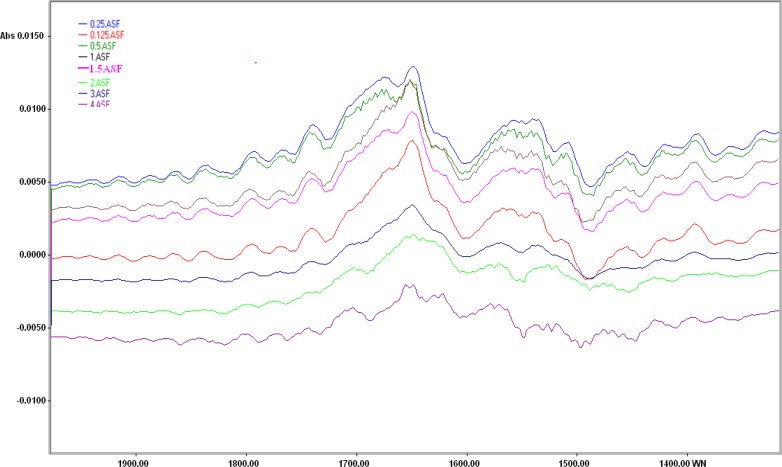
Biochemical index spectra of HepG2 cells after 24 hours exposure to the different concentrations of cisplatin in the FTIR spectral region of 1800-1200 cm^-^

Interpretation of spectrum from raw cellular FTIR spectra is hard and misses lots of alterations. To solve this problem, biochemical index spectrum (BIS) was calculated as difference between the spectra of cells exposed to different cisplatin concentrations and those cells maintained in normal saline. As the concentration of cisplatin increased, the intensities of a positive peak at about 1648 cm^-1^and a negative peak at about 1490 cm^-1^ were decreased (Figure 3). 

*Data analysis*

**Figure 4:**
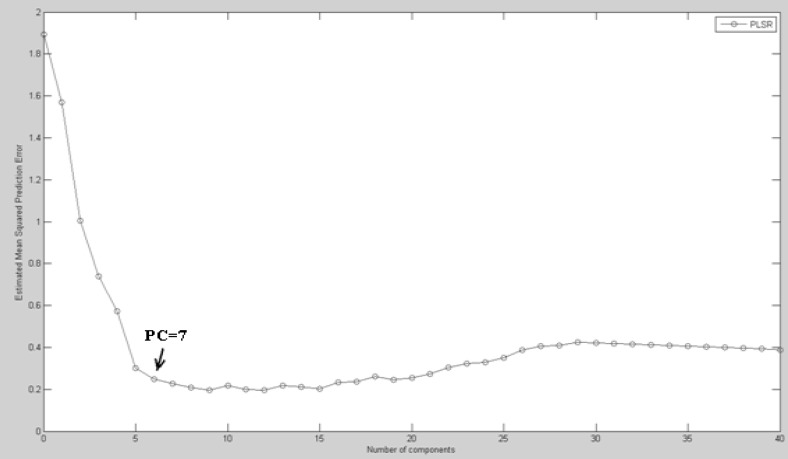
Estimated mean squared prediction errors of cross-validation FTIR response using PLS analysis.

FTIR data of **˝**Biochemical Index spectrum**˝ **(BIS) for different concentrations of cisplatin were sorted randomly into 20 different data sets (numbered 1 to 20) each composed of 40 training variables and 16 testing variables. The 20 models were analyzed with PLS analyzing to predict pattern for cisplatin toxicity. To choose an optimized number of latent variables (LVs) or principal components (PCs), we examined the mean squared prediction errors between the measured and the predicted responses with increasing numbers of LVs for each concentration of cisplatin. When BIS matrices were used to predict the effective concentration of cisplatin, the mean squared prediction errors decreased, while that was minimized with just 7 LVs for the PLS model (Figure 4).

In order to evaluate the performance of the models two statistical factors are used: the root mean square error (RMSE) and correlation coefficient (R^2^) values which are derived in statistical calculation of observations in model output predictions, defined as:

$\mathrm{RMSE 9t})=\sqrt[{2}]{\frac{1}{n}{\sum_{i=1}^{n}\left(W_{0}(t\right)-W_{p}(t))}^{2}}$

$R^{2}=1-\frac{\sum_{i=1}^{n}{(W}_{0}(t)-{W_{p}(t))}^{2}}{\sum_{i=1}^{n}{(W}_{0}(t)-{W_{0}^{'}(t))}^{2}}$

Where w_o_ is the observed values of cisplatine concentration for type t, w_o_ is the average of cisplatin concentration for type t, and w_p_ is the predicted value of cisplatin concentration for type t (30).

After selecting the optimized number of PCs for each model, we examined the squared Pearson correlation coefficient, *R*^2^, between exposed cisplatin and the predictions (Table 2). When the model is performed for the training dataset in present investigation, cisplatin concentration for each experiment in the testing dataset is predicted in turn using the learned rules derived from the dataset in model training procedure. Comparison of the 20 PLS models indicates a high correlation in all predictions for data sets of total FTIR wave number (Seri1; 1000-3000 cm^-1^) in training and testing data. Partly correlation was found for the range of 2000-3000 cm^-1 ^while there is no suitable correlation in other segmentation of FTIR data (*R*^2^ ranging from 0.3 to 0.77). 

**Table 2:** Prediction of accuracy in the training and the testing models using PLS calculations as described in methods.

	**R** ^2 ^ **for training model**					**RMSE for training model**			**R** ^2^ **for testing ** **model**	**RMSE for testing model**
Seri1	Models trained with variables in 1000-3000 cm^-1^									
	1	0.9144				0.1888			0.9807	0.0094
	2	0.9128				0.1699			0.9762	0.03
	3	0.9387				0.11			0.9188	0.14
	4	0.8515				0.179			0.9972	0.008
Seri2	Models trained with variables in 3000-2500 cm^-1^									
	5		0.8325			0.375			0.9869	0.006
	6		0.8644			0.26			0.9745	0.033
	7		0.8409			0.24			0.8215	0.018
	8		0.8014			0.2			0.9937	0.01
Seri3	Models trained with variables in 2500-2000 cm^-1^									
	9			0.8964		0.23			0.9329	0.032
	10			0.9124		0.17			0.9970	0.0177
	11			0.9387		0.1143			0.9181	0.14
	12			0.8518		0.1795			0.9972	0.008
Seri4	Models trained with variables in 1500-2000 cm^-1^									
	13			0.4818		1.14			0.7102	0.14
	14			0.4172		1.12			0.9716	0.036
	15			0.5969		0.75			0.7981	0.348
	16			0.3224		0.82			0.9907	0.027
Seri5	Models trained with variables in 1000-1500 cm^-1^									
	17				0.7	0.66			0.9351	0.03
	18				0.7700	1.36			0.5326	0.6
	19				0.7515	0.46			0.9165	0.144
	20				0.6324	0.44			0.9618	0.113

This work was based on the hypotheses that chemical toxicity in cells induces very changes in the cell biomolecules and FTIR may be capable of detecting these variations. The eight concentration of cisplatin in hepG2 cells induced different levels of mortality where was detectable through the FTIR spectra.

Binding to DNA is believed to be the main cytotoxicity action of cisplatin. Direct platinum binding to guanine (N7) and thymine (O_2_) were appeared in cellular DNA bands at 1.5 µg/mL of cisplatin exposure and from proteins were appeared in the spectral patterns of cells exposed to higher concentration of cisplatin at 2 µg/mL. Spectral change for proteins and nucleic acid bands is critical point in the ability of FTIR for highlighting molecular changes in cisplatin toxicity. It is estimated that cisplatin first interact with DNA and then with the proteins since DNA spectral alterations occur from 1.5 µg/mL, while protein spectra remain unchanged until above 2 µg/mL. 

Biomonitoring involves the use of molecular markers as signaling indicators for the exposure of living organisms to chemicals. Therefore, biological monitoring through the analysis of cells, tissues, or body fluids of exposed species may lead to the identification of potentially hazardous exposures before when the symptoms appear. Exposure limits might then be established to minimize significant health risks (31). Biological monitoring in cells requires the prediction of chemicals effective concentration for cell components with a suitable organized bioassay. Here, we are introducing a good correlation between biochemical index of FTIR spectrum and its corresponding cisplatin cytotoxic concentrations. However, risk assessment is conventionally based on the estimation of administered dose or human exposure to drugs and chemicals in spatial site (32) . Our results have presented that PLS is a good model for the prediction of toxic concentrations of cisplatin on cells using alterations in FTIR spectrum in the range of 1000-3000 cm^-1^. Cisplatin ability to interact with the different components of cells further increases the possibility of FITR spectroscopy application as a biological monitoring tool. However, one limitation of this study is the estimation of the toxicity patterns with different prediction models such as artificial neuronal network.

Many factors may affect the outcome of chemical exposure and toxicity outcome. Partial least square regression of FTIR data offers the advantage of a fast and reproducible procedure, which can be used for direct prediction of effective concentration for toxic agents in a bioassay procedure. 

## Conclusion

The interactions between chemicals and macromolecules in the cells are an important point for toxicological paradigms. Regarding this study, the step by step of toxicity stress in cell line is specified thorough FTIR spectroscopy analysis. HepG2 cell line was exposed with different concentration of cisplatine where hepatica cell is preferred for modeling of toxicity stress. In this study, all cisplatine detectable alteration was seen in DNA, amid and lipid bands of cells. Biological monitoring in cells requires the prediction of chemicals effective concentration for cell components with a suitable organized bioassay. Here, we are introducing a good correlation between biochemical index of FTIR spectrum and its corresponding cisplatin cytotoxic concentrations. In view of future, one of the advantages of this model is to predict the toxic concentration in animal exposure as well as the estimation of the bioaccumulation of chemicals in chronic exposure using PLS calculations on FTIR data.

## References

[1] Corte L, Rellini P, Roscini L, Fatichenti F, Cardinali G (2010). Development of a novel, FTIR (Fourier transform infrared spectroscopy) based, yeast bioassay for toxicity testing and stress response study. Analytica Chimica. Acta..

[2] Yang J, Yen HE (2002). Early salt stress effects on the changes in chemical composition in leaves of ice plant and Arabidopsis. A fourier transform infrared spectroscopy study. Plant Physiol..

[3] Kole MR, Reddy RK, Schulmerich MV, Gelber MK, Bhargava R (2012). Discrete frequency infrared microspectroscopy and imaging with a tunable quantum cascade laser. Analytical. Chem..

[4] Bellisola G, Cinque G, Vezzalini M, Moratti E, Silvestri G, Redaelli S, Passerini CG, Wehbe K, Sorio C (2013). Rapid recognition of drug-resistance/sensitivity in leukemic cells by Fourier transform infrared microspectroscopy and unsupervised hierarchical cluster analysis. Analyst.

[5] Ashtarinezhad A, Shirazi H, Vatanpour H, Mohamazadehasl B, Panahyab A, Nakhjavani M (2014). FTIR-Microspectroscopy Detection of Metronidazole Teratogenic Effects on Mice Fetus. Iran J Pharm Res..

[6] Portenier I, Waltimo T, Ørstavik D, Haapasalo M (2005). The susceptibility of starved, stationary phase, and growing cells of enterococcus faecalis to endodontic medicaments. J. Endodontics..

[7] McCann MC, Defernez M, Urbanowicz BR, Tewari JC, Langewisch T, Olek A, Wells B, Wilson RH, Carpita NC (2007). Neural network analyses of infrared spectra for classifying cell wall architectures. Plant Physiol.

[8] German MJ, Hammiche A, Ragavan N, Tobin MJ, Cooper LJ, Matanhelia SS, Hindley AC, Nicholson CM, Fullwood NJ, Pollock HM (2006). Infrared spectroscopy with multivariate analysis potentially facilitates the segregation of different types of prostate cell. Biophysical. J..

[9] Modaresi MS, Faramarzi MA, Soltani A, Baharifar H, Amani A (2014). Use of ArtificialNeural Networks to Examine Parameters Affecting the Immobilization of Streptokinase in Chitosan. Iran J Pharm Res.

[10] Ellis DI, Broadhurst D, Kell DB, Rowland JJ, Goodacre R (2002). Rapid and quantitative detection of the microbial spoilage of meat by Fourier transform infrared spectroscopy and machine learning. Applied Environment. Microbiol..

[11] Van Gestel IT, Baesens B, Garcia IJ, Van Dijcke P (2003). A support vector machine approach to credit scoring. Citeseer.

[12] Wu Y, Johnson GL, Gomez SM (2008). Data-driven modeling of cellular stimulation, signaling and output response in RAW 264.7 cells. J. Molecular Signaling.

[13] Rabik CA, Dolan ME (2007). Molecular mechanisms of resistance and toxicity associated with platinating agents. Cancer Treatment Rev.

[14] Polak M, Plavec J, Trifonova A, Földesi A, Chattopadhyaya J (1999). The change in the electronic character upon cisplatin binding to guanine nucleotide is transmitted to drive the conformation of the local sugar-phosphate backbone—a quantitative study. J. Chem. Soc..

[15] Rjiba-Touati K, Ayed-Boussema I, Belarbia A, Achour A, Bacha H (2012). Recombinant human erythropoietin prevents cisplatin-induced genotoxicity in rat liver and heart tissues via an antioxidant process. Drug Chem. Toxicol..

[16] Harrington CF, Le Pla RC, Jones GD, Thomas AL, Farmer PB (2010). Determination of cisplatin 1, 2-intrastrand guanine−guanine dna adducts in human leukocytes by high-performance liquid chromatography coupled to inductively coupled plasma mass spectrometry. Chem. Res. Toxicol..

[17] Codony-Servat J, Gimeno R, Gelpi C, Rodriguez-Sanchez JL, Juarez C (1996). The two isoforms of the 90-kDalton nucleolus organizer region autoantigen (upstream binding factor) bind with different avidity to DNA modified by the antitumor drug cisplatin. Biochem. Pharmacol..

[18] Shirazi FH, Molepo JM, Stewart DJ, Ng CE, Raaphorst GP, Goel R (1996). Cytotoxicity, accumulation, and efflux of cisplatin and its metabolites in human ovarian carcinoma cells. Toxicol. Applied Pharmacol..

[19] Raymond E, Faivre S, Chaney S, Woynarowski J, Cvitkovic E (2002). Cellular and Molecular Pharmacology of Oxaliplatin1. Molecular Cancer Therapeutics.

[20] Chaney SG, Campbell SL, Bassett E, Wu Y (2005). Recognition and processing of cisplatin-and oxaliplatin-DNA adducts. Critical Rev. Oncol./Hematol..

[21] Burger KN, Staffhorst RW, de Vijlder HC, Velinova MJ, Bomans PH, Frederik PM, de Kruijff B (2002). Nanocapsules: lipid-coated aggregates of cisplatin with high cytotoxicity. Nature Med.

[22] Pope A, Chary R-R, Alexander DM, Armus L, Dickinson M, Elbaz D, Frayer D, Scott D, Teplitz H (2008). Mid-infrared spectral diagnosis of submillimeter galaxies. Astrophysical J.

[23] Westerhuis JA, Kourti T, MacGregor JF (1998). Analysis of multiblock and hierarchical PCA and PLS models. J. Chemometrics.

[24] Wold H (1985). Partial least squares. Encyclopedia Statistical Sci.

[25] Jangir DK, Tyagi G, Mehrotra R, Kundu S (2010). Carboplatin interaction with calf-thymus DNA: A FTIR spectroscopic approach. J. Molecular Structure.

[26] Kong L, Liu Z, Hu X, Liu S (2011). Interaction of polymyxin B with ds-DNA, and determination of DNA or polymyxin B via resonance Rayleigh scattering and resonance non-linear scattering spectra. Microchimica Acta.

[27] Nakamura T, Kelly JG, Trevisan J, Cooper LJ, Bentley AJ, Carmichael PL, Scott AD, Cotte M, Susini J, Martin-Hirsch PL (2010). Microspectroscopy of spectral biomarkers associated with human corneal stem cells. Molecular Vision.

[28] Zanier K, Ruhlmann C, Melin F, Masson M, Ould M'hamed Ould Sidi A, Bernard X, Fischer B, Brino L, Ristriani T, Rybin V (2010). E6 proteins from diverse papillomaviruses self-associate both in-vitro and in-vivo. J. Molecular Biol..

[29] Neault J, Tajmir-Riahi H (1998). Interaction of cisplatin with human serum albumin. Drug binding mode and protein secondary structure. Biochimica et Biophysica Acta.

[30] Antanasijević D, Pocajt V, Popović I, Redžić N, Ristić M (2013). The forecasting of municipal waste generation using artificial neural networks and sustainability indicators. Sustainability Sci.

[31] Snedeker S (2005). Tracking chemical exposures with biomonitoring. Changes.

[32] Lucier GW, Thompson CL (1987). Issues in biochemical applications to risk assessment: When can lymphocytes be used as surrogate markers. Environment. Health Perspectives.

